# Frequency of Gastroparesis Symptoms in Patients With Type-2 Diabetes Mellitus at a Tertiary Care Hospital in Pakistan

**DOI:** 10.7759/cureus.44236

**Published:** 2023-08-28

**Authors:** Mohammad Shahzad Asif, Muhammad Khubaib Gul Khan, Muhammad Asad Nabeel, Tabin Ashfaq, Areeqa Nasir, Rai Muhammad Abdullah, Muhammad Ahmad Faraz Kareem

**Affiliations:** 1 Medicine, University College of Medicine and Dentistry, Lahore, PAK

**Keywords:** vomiting, type-2 diabetes mellitus, nausea, gastroparesis, bloating

## Abstract

Background

Gastroparesis symptoms seem to affect many diabetes mellitus patients. Pakistan has a high prevalence of diabetes, with an estimated 33 million people living with the condition. This study aimed to investigate the frequency of gastroparesis symptoms in patients with type-2 diabetes mellitus (T2DM).

Methods

This cross-sectional study was conducted from April to May 2022 in the outpatient Department of Medicine at the University College of Medicine and Dentistry, Lahore, Pakistan. Patients of both genders aged between 18 and 75 years and having T2DM were analysed. Data regarding demographic information, medical history, BMI assessment, and symptoms related to gastroparesis, as measured by the Gastroparesis Cardinal Symptoms Index (GCSI) were collected at the time of enrolment. For gastroparesis, a diagnostic cutoff of ≥1.90 was considered as per GCSI.

Results

Of a total of 148 T2DM patients, 85 (57.4%) were females. The mean age was calculated to be 54.0±11.3 years, ranging between 23 and 75 years. There were 134 (90.5%) patients who were using metformin. The most common symptom was fullness, reported by 66 (44.6%), while nausea, bloating, early satiety, retching, and vomiting were noted in 61 (41.2%), 59 (39.9%), 55 (37.2%), 39 (26.4%), and 22 (14.9%) patients, respectively. The frequency of gastroparesis was found in 17 (11.5%) T2DM patients. Stratification of gastroparesis revealed a significant association with female gender (82.4% vs. 54.2%, p=0.0272).

Conclusion

The study found a high frequency of gastroparesis symptoms in patients with T2DM. Nausea and bloating were the most commonly reported symptoms, while vomiting was the least common. The prevalence of gastroparesis was significantly higher in the female gender.

## Introduction

Gastroparesis is a debilitating condition that affects the normal functioning of the stomach muscles, leading to delayed gastric emptying. It is a common complication of diabetes, with an estimated prevalence of up to 50% in patients having diabetes mellitus [1]. Gastroparesis can cause a variety of symptoms, including nausea, vomiting, bloating, early satiety, and abdominal pain, which can significantly impact the quality of life of affected individuals [2]. Gastroparesis is a complex disorder, and its pathogenesis is not yet fully understood. However, it is thought to involve a combination of factors, including autonomic neuropathy, impaired gastric motility, abnormal regulation of the pyloric sphincter, and disturbances in the enteric nervous system [3]. Gastroparesis can occur in individuals with diabetes due to the detrimental effects of elevated blood sugar levels on the nerves that regulate stomach muscle functions.

There is significant variability in the occurrence of gastroparesis among diabetic patients in different countries and populations. Literature reports a high prevalence of gastroparesis symptoms in patients with type 1 diabetes compared to type 2 diabetes mellitus (T2DM) [4]. Additionally, there exists some supporting evidence to indicate that the frequency of gastroparesis may be greater in particular ethnic groups, including African Americans and Hispanics, in comparison to Caucasians [5]. Not much data about the frequency of gastroparesis symptoms among diabetic patients in Pakistan are available. The population of Pakistan is estimated to have a substantial number of individuals living with diabetes, with approximately 33 million people affected - ranking the third highest globally [6]. Nevertheless, there is a lack of extensive research on the prevalence of gastroparesis within this specific population. According to a recently published study analyzing patients with T2DM, 44% of patients had gastroparesis symptoms [7].

Understanding the prevalence of gastroparesis in patients with diabetes in Pakistan is important for several reasons. Firstly, it can help identify the burden of this condition in the population and inform healthcare planning and resource allocation. Additionally, it has the potential to assist in the identification of specific patient subgroups that may have an increased likelihood of developing gastroparesis, thus allowing for the implementation of targeted screening and management approaches. Finally, it can provide valuable insights into the pathophysiology of gastroparesis in patients with diabetes in Pakistan. Our study aimed to assess the prevalence of gastroparesis among patients with diabetes who seek treatment at a tertiary care hospital in Lahore, Pakistan. Furthermore, we wanted to note the clinical characteristics exhibited by individuals diagnosed with gastroparesis. This study aimed to investigate the prevalence of gastroparesis in patients with T2DM.

## Materials and methods

This cross-sectional study was conducted from April to May 2022 in the outpatient Department of Medicine at the University College of Medicine and Dentistry, Lahore, Pakistan. Patients of both genders aged between 18 and 75 years and having T2DM were analysed. Patients with diabetic complications such as kidney damage (albumin excretion rate (>30 mg/24 hours) or diabetic foot ulcer were excluded. We also excluded patients who presented with other types of diabetes, severe comorbid diseases, and prior surgeries in the gastric region. A non-probability, convenient sampling technique was adopted. Informed and written consent were obtained from all participants, and the collected data were treated with strict confidentiality. Approval from the Institutional Ethical Committee was obtained (letter number: IEC/2022/256).

Data regarding demographic information, medical history, BMI assessment, and symptoms related to gastroparesis, as measured by the Gastroparesis Cardinal Symptoms Index (GCSI), were collected at the time of enrolment. The GCSI has three symptom subscales, including nausea/vomiting, postprandial symptoms/early satiety, and bloating. Patients were asked to describe their symptoms with no symptoms as zero score up to 5 as severe symptoms. For gastroparesis, a diagnostic cutoff of ≥1.90 was considered [8,9]. To calculate BMI, we used WHO standards where BMI≥30 kg/m² was considered obese, ranging between 18.5 and 24.9 was considered normal, and BMI<18.5 for underweight.

The data were analysed using Statistical Package for Social Sciences (SPSS), version 26.0 (IBM SPSS Statistics for Windows, Armonk, NY). Descriptive statistics, such as mean, range, standard deviation, number, and percentages, were calculated. Chi-square and independent sample t-tests were used for inferential statistics. To find statistical significance a p-value of <0.05 was set.

## Results

Of a total of 148 T2DM patients, 85 (57.4%) were females. The mean age was calculated to be 54.0±11.3 years, ranging between 23 and 75 years. There were 134 (90.5%) patients who were using metformin. Characteristics of all T2DM patients analysed in this study are shown in Table 1.

**Table 1:** Characteristics of patients

Characteristics		Frequency (%)
Gender	Male	63 (42.6%)
	Female	85 (57.4%)
Body mass index	Underweight	1 (0.7%)
	Normal	18 (12.2%)
	Overweight	52 (35.1%)
	Obese	77 (52.0%)
Duration of diabetes (years)	≤5	43 (29.1%)
	6-10	51 (34.4%)
	11-15	24 (16.2%)
	>15	30 (20.3%)
Treatment	Metformin	134 (90.5%)
	Other oral hypoglycemic agents	93 (62.8%)
	Insulin	59 (39.9%)
Hypertension		34 (23.0%)

The most common symptom was fullness, reported by 66 (44.6%), while nausea, bloating, early satiety, retching, and vomiting were noted in 61 (41.2%), 59 (39.9%), 55 (37.2%), 39 (26.4%), and 22 (14.9%) patients, respectively. Table 2 shows the severity of gastroparesis symptoms in T2DM patients.

**Table 2:** Severity of gastroparesis symptoms in T2DM patients (N=148)

Symptoms	Equable (%)	Mild (%)	Moderate (%)	Severe (%)	Intense (%)	None (%)
Vomiting	6 (3.7)	8 (5.4)	6 (3.7)	1 (0.6)	1 (0.6)	126 (85.1)
Nausea	20 (13.5)	24 (16.2)	10 (6.7)	6 (3.7)	1 (0.6)	87 (58.1)
Fullness	16 (10.8)	25 (16.8)	19 (12.8)	5 (3.3)	1 (0.6)	81 (55.4)
Retching	12 (8.1)	14 (9.4)	6 (3.7)	6 (3.7)	1 (0.6)	109 (73.6)
Early Satiety	17 (11.4)	20 (13.5)	12 (8.1)	5 (3.3)	1 (0.6)	93 (62.8)
Bloating	13 (8.78)	22 (14.8)	16 (10.8)	5 (3.3)	3 (2)	89c (60.1)

The frequency of gastroparesis symptoms was found in 17 (11.5%) T2DM patients. Stratification of gastroparesis revealed a significant association with female gender (82.4% vs. 54.2%, p=0.0272). There was no significant association of gastroparesis with age (p=0.607), BMI (p=0.8526), duration of diabetes (p=0.0597), or hypertension (p=0.6033), as shown in Table 3.

**Table 3:** Stratification of gastroparesis with respect to gender, age, BMI, duration of diabetes, and hypertension (N=148)

Characteristics		Gastroparesis		P-value
		Yes (n=17)	No (n=131)	
Gender	Male	3 (17.6%)	60 (45.8%)	0.0272
	Female	14 (82.4%)	71 (54.2%)	
Age in years (Mean±SD)		55.1±12.0	53.9±11.2	0.6807
BMI	Underweight	-	1 (0.8%)	0.8526
	Normal	3 (17.6%)	15 (11.5%)	
	Overweight	5 (29.4%)	47 (35.9%)	
	Obese	9 (52.9%)	68 (51.9%)	
Duration of diabetes (years)	≤5	2 (11.8%)	41 (31.3%)	0.0597
	6-10	4 (23.5%)	47 (35.9%)	
	11-15	4 (23.5%)	20 (15.3%)	
	>15	7 (41.2%)	23(17.6%)	
Hypertension		5 (29.4%)	31 (23.7%)	0.6033

## Discussion

Our study found a frequency of gastroparesis symptoms at 11.5% in patients with T2DM attending a tertiary care hospital. This is a significant finding, considering the high burden of T2DM in Pakistan and the limited research on the prevalence of gastroparesis in this population. Our findings are consistent with a study done by Almogbel et al. from Saudi Arabia [10], where the authors reported that 10.8% of T2DM patients had clinical symptoms of gastroparesis. Another study by Alanazi et al. showed that the overall prevalence of gastroparesis symptoms as per GCSI in T2DM patients was 11.8% [11]. A study done by Kofod-Andersen et al. found the prevalence of gastroparesis in T2DM to be 9.8% [9]. In specialized centres, the literature has reported the prevalence of gastroparesis in T2DM between 10% and 30% [12-14]. The variations in the prevalence of gastroparesis in T2DM patients could be attributed to differences in study participants, different diagnostic criteria, and variations in the quality and care of T2DM.

Our study also highlights the importance of using a validated tool, such as the GCSI. The GCSI has been shown to be a reliable and valid tool for measuring gastroparesis symptoms and has been used in other studies [8-12] The present study noted that fullness was the most frequent symptom noted in 11.5% of patients. Asghar et al. found a 44% prevalence of gastroparesis symptoms in their study, which was quite high. Nausea was present in 33.1% of the patients, and retching and stomach fullness were present in 14.2% and 44.5% of participants, respectively [7]. One of the important findings of the present research was that the female gender had a significant association with gastroparesis. These findings are aligned with what has already been described in the literature [10,15]. Female gender is highlighted as a significant predictor of gastroparesis in T2DM, but the exact pathophysiological mechanism behind this is still unknown [16,17]. Some researchers have pointed toward estrogen-level variations as delayed gastric emptying is more prevalent among pre-menopausal females using oral contraceptives and post-menopausal females who received hormone therapy [18-20]. It is also known that functional gastrointestinal disorders are relatively more prevalent among females than males [21]. Another study found that gastrointestinal disorders were more prevalent in women compared to men, which is also the finding of our study [22]. Our study showed that hypertension was more prevalent in patients with diabetic gastroparesis, which is in accordance with the results of a previous study [23]. No exact mechanism showing the relationship of hypertension with gastroparesis symptoms is in view and needs further research.

The findings of this study suggested that gastroparesis symptoms are prevalent among T2DM patients, with nausea, fullness, and bloating being the most common symptoms. The data also indicated that the female gender may be a risk factor for the development of gastroparesis symptoms in T2DM patients. These findings can help healthcare providers identify and manage gastroparesis symptoms in T2DM patients, leading to improved quality of life in the affected patients.

Limitations

Although our study provides insights into gastroparesis prevalence in T2DM patients attending a tertiary care hospital in Lahore, it has limitations. The small sample size involved in this study meant that the generalizability of our findings was not endorsed. Neither gastric imaging nor studies were performed to rule out any possible mechanical obstruction. We could not evaluate glycemic control and its possible association with gastroparesis symptoms. Future studies with larger sample sizes and more diverse populations are needed to confirm our results. Furthermore, the prevalence of gastroparesis may differ in patients with type 1 diabetes or other forms of diabetes and in other regions of Pakistan. Our findings contribute to understanding the burden of gastroparesis in Pakistan and can inform healthcare planning and research. Larger studies are needed to develop targeted screening and management strategies for gastroparesis in patients with diabetes in Pakistan.

## Conclusions

The study found a high prevalence of gastroparesis symptoms in patients with T2DM. Nausea and bloating were the most commonly reported symptoms, while vomiting was the least common. The prevalence of gastroparesis symptoms was significantly higher in the female gender. Early detection and management of these symptoms are crucial in the prevention of complications associated with gastroparesis. Healthcare providers should be vigilant in screening and managing gastroparesis symptoms in patients with T2DM. A detailed, more comprehensive multicentre study is required for a better understanding of the disease.
